# Prevalence of chronic fatigue syndrome in metropolitan, urban, and rural Georgia

**DOI:** 10.1186/1478-7954-5-5

**Published:** 2007-06-08

**Authors:** William C Reeves, James F Jones, Elizabeth Maloney, Christine Heim, David C Hoaglin, Roumiana S Boneva, Marjorie Morrissey, Rebecca Devlin

**Affiliations:** 1 Chronic Viral Diseases Branch, Coordinating Center for Infectious Diseases, Centers for Disease Control and Prevention, Atlanta, GA, USA; 2 Department of Psychiatry & Behavioral Sciences, Emory University School of Medicine, Atlanta, GA, USA; 3 Abt Associates Inc, Cambridge, MA, USA; 4 Abt Associates Inc, Chicago, IL, USA

## Abstract

**Background:**

Chronic fatigue syndrome (CFS) is a debilitating illness with no known cause or effective therapy. Population-based epidemiologic data on CFS prevalence are critical to put CFS in a realistic context for public health officials and others responsible for allocating resources.

**Methods:**

Based on a random-digit dialing survey we ascertained CFS cases and controls to estimate the prevalence of CFS in metropolitan, urban, and rural populations of Georgia. This report focuses on the 5,623 of 19,381 respondents ages 18 to 59 years old. Fatigued (2,438), randomly selected unwell not fatigued (1,429) and randomly selected well (1,756) respondents completed telephone questionnaires concerning fatigue, other symptoms, and medical history. Subsets of those identified by interview as having CFS-like illness (292), chronic unwellness which was not CFS-like (268 – randomly selected), and well subjects (223, matched to those with CFS-like illness on sex, race, and age) completed a clinical evaluation.

**Results:**

We estimated that 2.54% of persons 18 to 59 years of age suffered from CFS. There were no significant differences in prevalence of CFS between metropolitan, urban or rural populations or between white and black residents of the three regions. However, there were significant differences in female-to-male ratios of prevalence across the strata (metropolitan female: male 11.2 : 1, urban 1.7 : 1, rural 0.8 : 1).

**Conclusion:**

We estimated that 2.54% of the Georgia population suffers from CFS, which is 6- to 10-fold higher than previous population-based estimates in other geographic areas. These differences may reflect broader screening criteria and differences in the application of the case definition. However, we cannot exclude the possibility that CFS prevalence may be higher in Georgia than other areas where it has been measured. Although the study did not identify differences in overall prevalence between metropolitan, urban, and rural Georgia populations, it did suggest the need for additional stratified analyses by geographic strata.

## Background

Chronic fatigue syndrome (CFS) is a complex medical and public health problem that is associated with severe personal suffering and loss. The median duration of illness is 7 years, a quarter of those with the illness are unemployed or receiving disability, and the average affected family forgoes approximately $20,000 in annual earnings and wages [1]. Yet, fewer than 20 percent have received medical care for CFS [2,3]. Despite more than 3,000 articles in the peer-reviewed medical literature, the pathophysiology of CFS is not well understood. There are no diagnostic laboratory abnormalities or clinical tests. There is no public health control or prevention strategy for CFS.

An understanding of the prevalence and distribution of CFS in the general population is fundamental to focusing etiologic research, estimating the effects of CFS on quality of life and productivity, and devising control and prevention strategies. The few studies which have estimated the prevalence of CFS in defined populations [4,3,6] reported prevalence estimates for CFS in adults to be between 0.24% [3] and 0.42% [5], and prevalence for CFS-like illness between 0.25% [4] and 1.67% [6]. These studies used different sampling, screening, and evaluation strategies; so, their results are not strictly comparable. They also identified relatively small numbers of persons with CFS, and thus they lack statistical power. However, all four studies documented that CFS disproportionately affects women, is more common in the economically disadvantaged, and affects racial/ethnic minorities at rates equal to or greater than those of whites.

We conducted the survey herein reported to estimate the prevalence of CFS in racial/ethnic groups representative of defined metropolitan, urban, and rural populations. The primary objective was to obtain information that could be used as a basis for the development and evaluation of a control strategy for CFS. The study also rectified two major weaknesses of previous studies. First, previous studies have screened the population for individuals with fatigue and then evaluated them for CFS. Although fatigue is central to CFS, focusing on fatigue ignores other important dimensions of the illness such as impaired memory or concentration, unrefreshing sleep, and bodily pain. For many persons who suffer from CFS, these symptoms, rather than fatigue, constitute the primary complaint. In addition, a household informant may not be familiar with specific symptoms but can identify whether someone is generally unwell or not. Thus, rather than limiting the initial screening stage to fatigued and non-fatigued persons, this survey cast a broader screening net, utilizing household informants to identify unwell (fatigue, problems with memory/concentration, unrefreshing sleep, or pain) members of these populations. We then conducted detailed evaluations of participants who were identified by a household informant as unwell to further identify those with CFS. Second, previous studies have not defined CFS in a rigorous reproducible manner (i.e., they did not use validated and standardized instruments to measure fatigue, impairment, and accompanying symptoms) [7]. To address this deficit, we used validated and standardized instruments to define CFS according to criteria of the 1994 case definition [8].

## Methods

The CDC Institutional Review Board, as required by Department of Health and Human Services regulations, approved the study. All participants were volunteers who gave informed consent. The study was conducted in English. Non-English speaking respondents were not included.

### Study Design

#### Metropolitan, urban, rural

The definitions of metropolitan, urban and rural geographic strata are complex. The U.S. Office of Management and Budget defines several categories of metropolitan statistical areas according to specific standards. In general, metropolitan areas contain at least a million residents living in a core area (i.e., central city), together with adjacent communities that have a high degree of economic and social integration with that core. Atlanta, with approximately 4 million residents, is a metropolitan city. The Census Bureau defines urban and rural areas independently of OMB's classification. Urban and rural can occur inside of and outside of metropolitan areas. Typically, settled areas of 2,500 or more are considered to be urban and the remainder rural. Based on these definitions, we determined that the cities of Macon and Warner Robins (with populations of 300,000 and 48,000, respectively) were urban. For this study, we considered the counties surrounding Macon and Warner Robins to be rural.

#### Survey in general

The survey was conducted between September 2004 and July 2005. It included residents of three areas of Georgia: metropolitan (Atlanta – Fulton and DeKalb counties), urban (Macon – Bibb County and Warner Robins in adjacent Houston County), and rural (10 counties surrounding Bibb County – Houston -excluding Warner Robins, Baldwin, Bleckley, Crawford, Jones, Macon, Monroe, Peach, Twiggs, and Wilkinson). The survey used the same strategy as previously reported CDC population surveys of CFS [7,6]. We used list-assisted random-digit dialing [9] and an advance letter [10] to contact households containing persons aged 18–59 years in the three population strata.

#### Telephone screening interviews

In contrast to our previous studies, which screened households for fatigue, in this study we modified the initial screening interview to cover a broader range of CFS defining symptoms. In brief, the screening interview asked a household informant (=> 18 years) to report the age, sex, ethnicity and health status of each household member aged 18 and older and to identify ***unwell ***household members, who the informant noticed to have at least one of the CFS defining symptoms (fatigue, cognitive impairment, unrefreshing sleep, muscle pain, joint pain, sore throat, tender lymph nodes, or headache) for ≥ 1 month, and ***well ***residents, who had none of these problems for => 1 month.

#### Detailed telephone interviews

Household residents between 18 and 59 years of age who were identified by the informant as unwell with fatigue, randomly selected persons identified as unwell without fatigue (i.e., identified with cognitive impairment, unrefreshing sleep, muscle pain, joint pain, sore throat, tender lymph nodes, or headache), and a random sample of people identified as well were asked to complete a detailed telephone interview. The detailed interview covered fatigue status and duration, other symptoms, race ("What race do you consider yourself to be? Please note that you may choose more than one option. White, black of African American, Asian, American Indian or Alaskan Native, native Hawaiian or other Pacific islander"), self-identified Hispanic/Latino or Spanish origin or descent, other demographic characteristics, and medical history. Based on their responses to the detailed interview, respondents were classified as: 1) having a medical or psychiatric condition considered exclusionary for CFS [7]; 2) having CFS-like illness if they reported severe fatigue lasting 6 months or longer that was not alleviated by rest, that caused substantial reduction in occupational, educational, social or personal activities, and that was accompanied by at least 4 of the CFS case defining symptoms [11]; 3) being chronically unwell (reporting any of the CFS defining symptoms) with or without fatigue);, 4) or being well.

#### Clinical evaluation

All respondents between 18 and 59 years who had no exclusionary conditions per interview, and were classified as having a ***CFS-like ***illness were invited for a one-day clinical examination to further investigate exclusionary medical and psychiatric conditions. We also invited a similar number of randomly selected participants identified with ***chronic unwellness ***(at least six months of unwellness with or without fatigue but not CFS-like). Finally, we invited ***well ***participants, matched to the CFS-like on geographic stratum, sex, race/ethnicity and age (within 3 years), for a one-day clinical examination. Those from the urban or rural areas attended a clinic in Macon, and those from Atlanta attended a similar clinic in Atlanta. No more than 4 participants attended a clinic on any day, and appointments were staggered for optimal flow. Clinic staff with responsibilities for examinations was not aware of participants' telephone interview responses or classification. The authors and CDC CFS Research Program staff attended clinics on a regular basis to assess operations.

#### Case definitions

##### Telephone interview

Study participants who underwent a detailed telephone interview and met criteria of the 1994 CFS case definition [11] were classified as ***CFS-like***. In brief, criteria for classification as CFS-like on telephone included persistent or relapsing fatigue of at least 6 months' duration; the fatigue was not relieved by rest and caused substantial reduction in previous levels of occupational, educational, social, or personal activities. Exclusionary conditions included self-reported medical or psychiatric conditions that could cause the fatigue. Finally, the medically/psychiatrically unexplained fatigue must have been accompanied by at least 4 of the 8 CFS case defining symptoms [11]: 1) unusual post-exertional malaise; 2) unrefreshing sleep; 3) impaired memory or concentration; 4) headaches; 5) muscle pain; 6) multi-joint paint without swelling or redness; 7) sore throat; 8) tender cervical/axillary lymph nodes. CFS-like subjects differ from CFS by not having been evaluated clinically in the study.

##### Clinic

The objective of the clinical evaluation was to classify participants' clinical status and diagnose exclusionary medical and psychiatric conditions. As recommended by the International CFS Study Group [7], participants were classified as ***CFS***, unexplained chronic illness not meeting criteria for CFS (termed ***ISF***), or ***well ***by using standardized reproducible criteria for measuring specifics of the 1994 case definition [8]. We used the Multidimensional Fatigue Inventory (MFI) [12] to assess fatigue status. For classification as ***CFS***, those with a score => well-population medians on the general fatigue **or **reduced activity scales of the MFI were considered to meet fatigue criteria of the 1994 case definition. Functional impairment was assessed by the medical outcomes survey short form-36 (SF-36) [13]. For classification as ***CFS***, those with a score =< 25^th ^percentile of population norms in the physical function **or **role physical, **or **social function, **or **role emotional subscales of the SF-36 were considered to have substantial reduction in activities as specified in the 1994 definition. Finally we used the CDC Symptom Inventory (SI) [14] to evaluate occurrence, frequency and severity of the 8 CFS-defining accompanying symptoms. The SF-36, MFI and SI domain scores require complete data for the subscales. We imputed a zero value in the case of one-item non-response for subscales contributing to the relevant domains. For classification as ***CFS***, those reporting => 4 case defining symptoms **and **who scored > 25 on the SI concerning frequency and severity of the 8 case defining symptoms [14] were considered to meet accompanying symptom criteria of the 1994 case definition. Participants who fulfilled some, but not all of these criteria were classified as ***ISF***. Those who met none of the criteria were considered to be ***well***. The MFI, SF-36 and SI are self-administered paper and pencil forms. A trained clinic supervisor reviewed forms and helped subjects complete missing or misunderstood portions.

To screen for medical conditions considered exclusionary for CFS [11,7], participants completed past medical history questionnaires and were requested to bring all their medications and supplements to the clinic. A licensed nurse practitioner or physician assistant reviewed subjects' past medical histories and medications to clarify omissions or discrepancies and also catalogued all medications. Relevant information was brought to the attention of the study physicians. A specifically trained licensed physician then performed a standardized physical examination [3]. The examination was expanded if there were any concerns. The examiner recorded a differential diagnosis. Blood and urine specimens were obtained for laboratory screening tests to identify possible underlying or contributing medical conditions as stipulated by the case definition [11,7]. Laboratory tests included a complete blood count with differential, c-reactive protein, alanine aminotransferase (ALT), SGPT, albumin, alkaline phosphatase, asparatate aminotransferase (AST), SGOT, total bilirubin, calcium, carbon dioxide, chloride, creatinine, glucose, potassium, total protein, sodium, urea nitrogen BUN, antinuclear antibodies, rheumatoid factor, TSH, free T4, and urinalysis.

To screen for psychiatric conditions considered exclusionary for CFS [11,7], a trained and experienced licensed psychiatric social worker, clinical psychologist, psychiatric nurse practitioner or certified psychiatric research nurse administered the research version of the SCID [15]. They underwent specific training for the SCID. Psychologists on the CDC CFS Research Program monitored their technique on a regular basis. The SCID included the screening module, mood episodes, psychotic symptoms, psychotic disorders, mood disorders, substance use disorders, anxiety disorders, somatoform disorders, eating disorders, and adjustment disorders.

A review committee of CDC and Emory University physicians and psychologists reviewed medical and psychiatric evaluations to determine the presence of medical and psychiatric conditions exclusionary for CFS. Members of the review committee were not aware of subjects' classification either on phone interview or in the clinic.

### Weighting

Prevalence estimates and statistical analyses utilized weighted data. The survey weights maintained (through the stages of the survey) the relation between the sample and the population in each geographic stratum, and they included several adjustments that are customarily employed to reduce bias. In the process of developing weights, one step adjusted for households that did not have telephones. To estimate the proportion of households that did not have a telephone in each of the three geographic strata, we analyzed data from the 5% public-use microdata samples (PUMS) of the 2000 Census. For the metropolitan stratum, the analysis used PUMS data from De Kalb and Fulton Counties. The other two strata, however, do not correspond exactly to geographic entities for which data are available in the PUMS. Thus, the analysis for the urban stratum used data from Bibb County; the rural stratum used data from a larger combination of counties that contained the counties of that stratum. The resulting estimates of the proportion of households that did not have a telephone were 1.68% in the metropolitan stratum, 3.66% in the urban stratum, and 6.35% in the rural stratum.

Adjustments for nonresponse on the detailed telephone interview and nonresponse on the clinical evaluation kept the categories of illness separate; thus, to the extent possible, respondents accounted for nonrespondents who belonged to the same illness category (and shared other key characteristics). The adjustment factor, applied to the weight of each respondent, equaled the ratio of the sum of the weights (at that stage) of respondents and nonrespondents to the sum of the weights of respondents. Another adjustment, at the household level, used data on interruptions in telephone service to compensate for the inability of the telephone survey to reach households that did not have telephone service.

Households completing the screening interview received a base sampling weight (64.4 in the metropolitan stratum, 6.4 in the urban stratum, and 4.9 in the rural Stratum) equal to the reciprocal of the probability that the household's telephone number was selected for the sample. Base weights were reduced for multiple residential telephone numbers in the household (either by a factor of 2 or a factor of 3) and adjusted for households that did not complete screening interviews (by a factor of 1.03 in each stratum), for numbers associated with undetermined residential status (by a factor of 2.4, 2.3, and 2.1, respectively), and for non-telephone households in the population (by a factor of 1.7, 2.2, and 2.3 for households that reported interruptions in telephone service and by a factor of 1.24, 1.38, and 1.04 for households that did not report interruptions) [16,17]. (The household weights ranged from 66 to 268 in the metropolitan stratum, from 7 to 34 in the urban stratum, and from 4 to 24 in the rural stratum.)

Subjects selected for detailed interviews received an initial interview weight equal to their household weight multiplied by the reciprocal of their probability of selection (the probability of selecting a household as a source of a subject ranged from 0.32 to 1.0 for unwell subjects and from 0.17 to 1.0 for well subjects; the probability of selecting an individual subject within the household was the reciprocal of the number of unwell, respectively well, persons in the household; subjects with prolonged fatigue were selected with certainty). Within each combination of stratum (metropolitan, urban, rural) and illness classification (fatigued, unwell, well), initial weights were adjusted for nonresponse on the detailed interview, within a total of 195 cells defined by sex, age, and race (the adjustment factors ranged from 1.05 to 2.25 and exceeded 2.0 in only 22 cells). A further adjustment in each stratum used an iterative form of post-stratification to bring the weighted totals into agreement with control totals from the 2000 Census on race and on the combination of sex and age. This process produced an interview weight for each subject who completed a detailed interview. (The interview weights ranged from 84 to 16,723 in the metropolitan stratum, from 5 to 822 in the urban stratum, and from 4 to 892 in the rural stratum.)

Each CFS-like subject who completed a clinical evaluation received a clinical-evaluation weight, which incorporated an adjustment for nonresponse on the clinical evaluation within stratum-specific cells defined by sex and age (over the 19 cells the adjustment factor ranged from 1.11 to 2.71). For chronically unwell subjects the clinical-evaluation weights incorporated a parallel adjustment for nonresponse (over the 14 cells the adjustment factor ranged from 1.52 to 2.61), preceded by an adjustment for selection of the subsample (by a factor of 3.02, 3.88, and 2.89 in the respective strata). (The clinical-evaluation weights ranged from 193 to 32,354 in the metropolitan stratum, from 8 to 1,787 in the urban stratum, and from 10 to 1,358 in the rural stratum.) Because they were selected for clinical evaluation only as a result of being matched to a CFS-like subject, well subjects did not have their own clinical-evaluation weight.

### Prevalence estimates

Within each stratum, prevalence was estimated using SUDAAN (SUDAAN: Research Triangle Institute, Research Triangle Park, NC) [18] software to calculate weighted percentages and obtain standard errors that reflected the sample design and survey weights. Prevalence estimates based on illness classifications derived from the detailed interviews used the data of all subjects who completed detailed interviews, and their respective interview weights.

In order to maintain the relation to the population, prevalence estimates based on illness classifications derived from clinical data used a combination of clinical data and interview data. For CFS-like and chronically unwell subjects who completed clinical evaluations, the data obtained from the clinical evaluations were used along with their clinical-evaluation weights. For subjects classified as CFS-like and chronically unwell who were not eligible for clinical evaluations, and also for all well subjects, the data obtained from detailed interviews were used along with their interview weights.

Because the matching process does not preserve a sampling-based connection with the population, clinical data from well subjects were not used in calculating prevalence estimates.

### Statistical analyses

Weighted χ^2 ^tests in SUDAAN were used to compare proportions of subjects diagnosed with CFS by demographic categories. P-values were calculated to evaluate the statistical significance of differences in CFS prevalence by age, sex, race, ethnicity, education and household income.

## Results

### Screening telephone interview

Overall, 105,000 telephone numbers were selected for a screening interview; 66,295 (63%) were ineligible because they belonged to businesses, were not working, or were cellular phones. Residential status could not be determined for 24,594 (23%) of the numbers – 2,136 (2%) because all attempts produced no contact, 4,258 (4%) because attempts reached only an answering machine, and 18,200 (17%) because of another outcome (primarily the person refused to participate before household status could be determined). A total of 2,864 numbers (3%) belonged to households where all residents were over the age of 59; these households were not eligible for the study. The remaining 11,247 numbers (11%) were residential and eligible for screening. We completed screening telephone interviews for 10,837 of the identified residential numbers (96% participation). Taking into account estimated numbers of age-eligible households among the telephone numbers for which residential status could not be determined and among the households for which screening was not completed, the response rate for the screening step was 79%. There were no important differences in response across the strata (range 76.1% to 81.4%).

The screening interviews enumerated 19,381 residents. Of these, 10,834 (56%) were identified by the household informant as well, 5,122 (26%) as unwell for at least a month but not fatigued, and 3,425 (18%) as unwell and fatigued for at least a month. We attempted to conduct detailed telephone interviews on all those who were unwell with fatigue, and 2,438 (71%) completed the detailed interview. We randomly selected 2,134 of those who were unwell not fatigued, and 1,429 (67%) completed detailed interviews; similarly, 1,756 (56%) of 3,113 randomly selected household members identified as well completed detailed telephone interviews. There were no important differences in detailed interview completion across the strata (range 66.8% to 72.6%)

### Telephone interview sample

Individuals' responses during the detailed telephone interview roughly mirrored the household informants' classification (Table 1). For example, 65% of those described as well by household informants during the screening interview, described themselves as 'well' during the detailed interview. Similarly, 69% of those described as unwell not fatigued by household informants, described themselves as unwell not fatigued. A smaller proportion (49%) of those who were initially described as unwell fatigued by household informants described themselves as unwell with fatigue during the detailed interview. Following the detailed telephone interview 1,513 respondents were classified as well, 1,803 as unwell for at least a month but not fatigued, 1,400 as unwell with fatigue, and 907 as CFS-like, meaning that they fulfilled all criteria of the 1994 case definition [11] on telephone interview.

**Table 1:** Screening Interview Classification

**Detailed Interview**	Well	Unwell not Fatigued	Unwell Fatigued
**Classification**	n = 1,756	n = 1,429	n = 2,438
Well (n = 1,513)	1,141 (65%)	242 (17%)	130 (5%)
Unwell not Fatigued (n = 1,803)	545 (31%)	982 (69%)	276 (11%)
Unwell Fatigued (n = 1,400)	57 (3%)	149 (10%)	1,194 (49%)
CFS-like (n = 907)	13 (1%)	56 (4%)	838 (34%)

(%) **indicates column percent**

Responses during the detailed telephone interview identified a self-reported medical or psychiatric explanation for the illness in 438 (48%) of CFS-like participants, 558 (40%) of those unwell with fatigue, and 429 (24%) of those who were unwell but not fatigued. Interestingly, 184 (12%) of participants classified as well reported medical and psychiatric conditions and these were in general the same as those reported by participants with CFS-like illness and those who were unwell.

### Clinic sample

We invited all 469 individuals with CFS-like illness and no medical/psychiatric exclusions for a clinical evaluation, and 292 (62%) agreed to participate. Those who did not come for a clinical evaluation were similar to those who did with regard to age, sex, income, and duration of illness. We randomly selected 505 from the 1,763 identified as ***chronically unwell ***(=> 6 months duration with or without fatigue) and invited them to clinic; 268 (53%) participated. Finally, we selected 641 interview participants classified as ***well ***(n = 481) or ***prolonged unwell***, i.e. unwell for 1 to 6 months duration (n = 160). These were matched to the ***CFS-like ***on sex, race, and age (within 3 years). A total of 163 participants classified as well and 60 who were classified as prolonged unwell completed clinical evaluations. As with telephone interviews, there were no important differences in clinical evaluation participation across the three strata, either overall or by classification status.

Clinical evaluation identified a medical or psychiatric exclusion in 26 (16%) of those classified as ***well ***based on their detailed telephone interview responses, 24 (40%) of those with ***prolonged unwellness***, 44 (30%) of the ***chronically unwell not fatigued***, 45 (38%) of the ***chronically unwell fatigued***, and 141 (48%) of the ***CFS-like***. The most frequent medical exclusions included previously undiagnosed thyroid disease (24% of the total), anemia (18%), uncontrolled diabetes (14%), autoimmune disease (11%), inflammatory disease (8%), heart disease (7%), arthritis (3%) and pulmonary disease (3%). Psychiatric exclusions encompassed alcohol or substance abuse (43%), melancholic depression (26%), bipolar disorder (19%), psychosis (7%), and anorexia/bulimia (5%). One subject enrolled with chronic unwellness and one CFS-like subject had incomplete medical or psychiatric evaluations and could not be classified.

Table 2 summarizes the relation between classification following detailed telephone interview and clinic classification among the 501 study subjects who did not have an exclusionary condition. One hundred-thirteen met criteria for ***CFS***, 264 had an unexplained illness (***ISF***), and 124 were ***well***. Interestingly, 53 (39%) of those who were classified as ***well ***based on their telephone interview data were classified as ***ISF ***(insufficient symptoms or fatigue for CFS) or ***CFS ***when evaluated in clinic, while 26 (15%) of those enrolled as ***chronically unwell ***based on telephone interview had no evidence of unwellness when evaluated in clinic.

**Table 2:** Clinic Classification of Participants with No Exclusionary Conditions

**Clinic Classification**			
**Detailed Interview**	Well	ISF	CFS
**Classification**	(n = 124)	(n = 264)	(n = 113)

Well (n = 137)	83 (61%)	53 (39%)	1 (1%)
Prolonged Unwell (n = 36)	15 (42%)	19 (53%)	2 (6%)
Unwell not Fatigued (n = 104)	20 (19%)	74 (71%)	10 (10%)
Unwell Fatigued (n = 74)	6 (8%)	52 (70%)	16 (22%)
CFS-like (n = 150)	0 (0%)	66 (44%)	84 (56%)

(%) **indicates row percent**

### Prevalence estimates

Table 3 summarizes prevalence of CFS in metropolitan, urban and rural populations according to demographic characteristics. Overall, 2.54% of the study populations had CFS; 83% reported gradual onset and 17% reported sudden onset of their illness. There were no statistically significant differences in prevalence of CFS among metropolitan, urban, and rural populations among women (p = .37). However, among men, CFS prevalence varied significantly among geographic strata, and was lowest in the metropolitan stratum (p = .038). In the metropolitan area, the CFS prevalence in women was 11.2 times that in men (p = .009), whereas in the urban and rural populations the female-to-male ratios of CFS prevalence were 1.7 and 0.8, respectively, and did not represent statistically significant differences.

**Table 3:** Prevalence of CFS (in percent) by Demographic Characteristics in the Three Populations

	**Metropolitan**	**Urban**	**Rural**
	% (SE)	% (SE)	% (SE)
**Overall prevalence**	2.55 (0.85)	2.48 (0.67)	2.66 (0.58)
**Sex**			
Female	4.70 (1.60)	3.10 (0.81)	2.40 (0.57)
Male	0.42 (0.37)	1.82 (1.08)	2.89 (0.97)
**Race**			
White	3.72 (1.71)	2.39 (0.70)	3.20 (0.72)
Black	1.78 (0.63)	2.71 (1.27)	1.71 (1.00)
**Ethnicity**			
Hispanic	21.21 (11.97)	7.02 (6.74)	0.70 (0.71)
Non-Hispanic	2.25 (0.82)	2.30 (0.64)	2.72 (0.59)
**Age**			
18–29	4.15 (2.28)	0.54 (0.29)	0.00
30–39	0.74 (0.43)	5.59 (2.40)	2.68 (1.06)
40–49	2.64 (1.34)	2.38 (0.83)	2.08 (0.77)
50–59	2.11 (1.13)	1.74 (1.09)	6.88 (2.17)
**Education**			
=< High School Graduation	0.00	1.02 (0.73)	2.48 (1.10)
High School Graduate	1.60 (1.24)	2.54 (1.02)	3.80 (1.34)
Technical or Some College	2.02 (1.01)	3.05 (1.60)	2.33 (1.15)
=> College Graduate	3.44 (1.47)	2.60 (1.25)	1.97 (0.72)
**Household Income**			
=< $20,000	4.85 (4.01)	1.87 (1.06)	1.87 (0.83)
$20,001–$40,000	3.43 (2.49)	2.89 (1.57)	4.72 (1.70)
>$41,000	1.60 (0.61)	2.79 (1.19)	2.25 (0.80)
**Poverty Level**			
=< $20,000	4.85 (4.01)	1.87 (1.06)	1.87 (0.83)
> $20,001	2.09 (0.80)	2.82 (0.95)	3.09 (0.79)

Overall, white and black adults had roughly similar rates (2.3% and 2.9%, respectively). Although Hispanic adults in metropolitan and rural populations had considerably higher prevalence than non-Hispanic, the numbers of Hispanics interviewed were low (23 in the metropolitan area, 38 urban, and 59 rural), and the differences were not statistically significant.

Age-specific prevalence of CFS differed among age categories in urban and rural populations (p = .041 and p = .0001, respectively), but not in metropolitan (p = .224). The rate was lowest among urban and rural adults aged 18–29 and highest among rural adults aged 50–59.

The prevalence of CFS was not significantly related to level of education in metropolitan, urban or rural strata (p = .129, p = .486, and p = .695, respectively). Similarly, CFS prevalence was not significantly related to household income in these strata (p = .741, p = .900, and p = .373, respectively).

## Discussion

This is the first published study, of which we are aware, that screened defined populations for unwellness and then used standardized, validated instruments to define CFS, unwellness, and wellness based on functional impairment, characteristics of fatigue, and frequency/severity of the 8 case defining symptoms [7]. Using this approach, we found 2.54% of the Georgia population to suffer from CFS, which was 10-fold higher than previous estimates in the population of Wichita (0.24%) [3] and 6-fold higher than estimated in the Chicago population (0.42%) [5]. We are aware of no other published population-based surveys of CFS. However, several studies have published estimates of CFS prevalence; although they cannot be directly compared to the 2 U.S. studies, their prevalence estimates serve to put the present study into a more complete perspective. A well-conducted survey of the Group Health Cooperative of Puget Sound estimated that between 0.75 and 0.27% of that HMO population had CFS [19]. A survey of primary care patients in England, published in 1997, estimated that 2.6% of that population met criteria for CFS [20]. Finally, analysis of data from the Australian National survey of Mental Health and Wellbeing estimated that 1.5% of the Australian adult population suffers from chronic neurasthenia (defined similarly to CFS) [21].

In part, the increased prevalence we estimated in Georgia reflects a difference in screening criteria. The Georgia survey screened for ***unwell ***(the core symptoms of CFS), whereas previous studies have screened only for fatigue. Our less restrictive approach allowed the inclusion of potential cases whom, although noted as unwell-not fatigued by a household informant, endorsed chronic fatigue upon detailed in-person interviewing. Sixty-nine people whom the household informant identified during the screening telephone interview as well or unwell without fatigue were classified as CFS-like based on their responses during detailed telephone interview (7.6% of all CFS-like). Further, 13 clinic participants classified as well or unwell without fatigue, based on their detailed telephone interview were diagnosed as CFS when evaluated clinically. In other words, 11.5% of subjects with CFS would not have been detected in previous studies that queried participants only for fatigue.

The 6- to 10-fold greater prevalence estimates also reflect application of more sensitive and specific measures of the CFS diagnostic parameters specified by the 1994 case definition. Previous prevalence estimates from population surveys and those based on patients attending clinics did not use validated standardized instruments to define CFS; rather they simply queried as to the presence or absence of fatigue, accompanying symptoms, and impairment. In 2003, the *International Chronic Fatigue Syndrome Study Group *published recommendations concerning application of the case definition [7]. They recommended the use of validated instruments to obtain standardized measures of the major symptom domains of the illness, and this study implemented those recommendations. The *Study Group *specifically recommended: 1) the SF-36, to measure functional impairment; 2) the Checklist Individual Strength or MFI, to obtain reproducible quantifiable measures of fatigue; 3) and the CDC Symptom Inventory to document toe occurrence, duration and severity of the symptom complex. The manner in which we applied the case definition in Georgia has been shown to detect about 3 times the number of CFS cases as verbatim application of the 1994 definition [8]. Of course, we cannot exclude the possibility that CFS prevalence may be higher in Georgia than Wichita and Chicago.

However, the manner in which we chose and applied subscales and their cutoffs from the SF-36 and MFI can be debated. We used the SF-36 physical function, role physical, social function and role emotional subscales to define an illness severe enough to "result in substantial reduction in previous levels of occupational, educational, social, or personal activities." [11]. In particular, we included the role emotional subscale to capture the relation between functional emotional impairment and reduced social and personal activities. We ascertained the onset and duration of fatigue during interview (the case definition requires => 6 months of fatigue that is of new or definite onset) and utilized the MFI general fatigue and reduced activity scales to define severe fatigue. We used stringent (i.e., =< 25^th ^percentile population norms on any of the 4 SF-36 scales) to define severe functional impairment. Numerous publications tabulate slightly different population norms and we chose those published by Quality Metric [22]. We are not aware of published population norms for the MFI, so we used the cut-offs established in a previous CDC study (=> than the median determined in Wichita) [8], which is more sensitive and less specific than the 25^th ^percentile SF-36 cutoff. There are no population norms for the Symptom Inventory, so we also used cutoffs applied in the previous study. Finally, population norms are not cast in iron and one might consider defining cut-offs specific to each population studied. In the end, we decided to use these cutoffs because we believe they make sense; because we used them in the Wichita study and can compare findings in similarly ill individuals; and, because others can replicate the findings if they use the same cutoffs and stratify their populations based on variations of the cutoffs.

The other important new finding is that, despite differences in demographic factors, CFS prevalence was similar in the metropolitan, urban, and rural populations we surveyed (about 2.5%). However, differences in sex-specific prevalence among the strata must be evaluated in more detail. The striking differences between female and male rates in the 3 strata may indicate risk effects of gender (a social construct) in distinction to sex (a biologic attribute). In addition, the high prevalence among those of Hispanic ethnicity in the metropolitan area bears further investigation in a study designed to include Spanish-speaking persons.

The final major finding concerns the high proportion of study participants in whom the survey documented previously undiagnosed medical or psychiatric conditions. Overall 48% of persons recruited for clinical evaluation because CFS-like illness was identified on phone interview had exclusionary medical or psychiatric conditions, which is similar to the occurrence of such illness in other studies [2,3]. Most of these exclusions are amenable to treatment if appropriately recognized. It is also important to note the relatively common occurrence of such conditions in people with other categories of unwellness identified during the phone interview. Indeed, 16% of respondents classified as well on the basis of interview had such exclusions identified. Further analyses will address whether the onset of psychiatric illness preceded or followed development of CFS-like illness.

Interpretation of the findings must consider obvious study weaknesses. First, like other telephone surveys, we faced challenges of nonresponse. Potential respondents can be lost in the processes of determining whether telephone numbers belong to households, completing screening interviews with household respondents, and maintaining contact with selected subjects to complete detailed interviews. In addition, some people are reluctant to schedule a full-day clinical examination. A random-digit-dialing survey that subsamples some types of subjects does not have a standard, single-number response rate. Taking into account the resolution of sampled telephone numbers (as residential or not), completion of screening interviews, and completion of detailed interviews, we calculated a response rate of 47.8% through the detailed interview. A further calculation, including the completion of clinical evaluations by subjects with CFS-like illness and chronically unwell subjects, produced a response rate of 27.5% through the clinic stage. Although nonresponse is always an appropriate subject for concern, detailed adjustments in the sampling weights often are able to mitigate its adverse effects. Thus, the adjustment for nonresponse on the detailed interview used a total of 195 cells, taking into account stratum, illness classification, sex, age, and race; post-stratification aligned respondents' weights with population totals; and the adjustment for nonresponse on the clinical evaluation used a total of 33 cells based on stratum, illness classification, sex, and age.

The second weakness concerns obtaining clinical information by telephone interview. Initial classification of subjects as well, unwell not fatigued, and unwell fatigued based on telephone interviews with household informants was confirmed by detailed interviews in 65%, 69% and 49% of subjects in those respective categories. Except for the unwell fatigued group, these levels of agreement between household informants and self-reports were similar to the 66% agreement detected in a study designed to examine concordance between self-reported health conditions and proxy information among adults => 65 years of age in the United Kingdom [23]. This degree of agreement was considered reasonable. The lower level of agreement detected among the unwell fatigued may be due to the relative nonspecificity of fatigue, compared to other symptoms of unwellness, including problems with memory/concentration, unrefreshing sleep, or muscle/joint pain.

Third, the study was conducted in standard 8^th ^grade-level English, which may have led to an under-sampling of ethnic minority groups (e.g., Spanish speaking). Prevalence of CFS was unusually high among the metropolitan and rural Hispanic populations and unusually low among rural Hispanic residents. This may reflect important ethnic differences in risk [2] and we weighted the sample to allow for ethnic differences. Most likely it reflects language-related misunderstanding of the questions and weighting cannot address this. It will be important to further evaluate the occurrence of and risk factors for CFS in English and non-English speaking metropolitan, urban, and rural Hispanic populations [24].

Fourth, the study utilized Atlanta (Fulton and DeKalb counties) to represent metropolitan Georgia, Macon and Warner Robins to represent urban Georgia, and the counties surrounding the urban area to represent rural Georgia. We did this for logistic reasons, and the results cannot a-priori be generalized to other populations. Indeed, populations of the 10 rural county seats varied from 587 to 19,000 (median 2,000), so several exceeded the Census Bureau definition of rural.

Finally, in spite of a rigorous case definition, CFS has no diagnostic markers or characteristic physical signs. Thus, CFS diagnosis is based on self-reported symptoms, disability and exclusion of known diseases. Therefore, potential misclassification of study subjects remains a concern, as CFS is clinically heterogeneous and likely represents more than one entity [25,26].

## Conclusion

In conclusion, this investigation suggests that 2.54% of the adult population of Georgia suffers from CFS. This figure is 6- to 10-fold higher than previous prevalence estimates and likely reflects improved screening methods and more sensitive and specific diagnostic criteria. However, this is the first study to measure CFS prevalence in Georgia, so we cannot rule out the possibility that CFS prevalence is higher in Georgia than other geographic areas where CFS prevalence has been reported. We did not find evidence for metropolitan, urban, rural differences in the prevalence of CFS, nor did we find differences in prevalence between white and black populations. These findings are important for public health officials, health care providers, and the public in general. The methodology and findings from this study should be of interest to those studying CFS and those with responsibilities for health care in other states in the US and in other countries.

## Competing interests

The author(s) declare that they have no competing interests.

## Authors' contributions

WCR was Principal Investigator of the study. WCR, EM, CH, RB, JFJ designed the study and wrote the protocol. DCH was responsible for sampling strategy and statistical analysis. WCR, CH, EM, RSB, JFJ supervised fieldwork. MM and RD supervised technical aspects of the study and supervised daily phone interviews and clinic operations. All authors contributed to preparation of the manuscript.
